# Single Domain Antibodies as New Biomarker Detectors

**DOI:** 10.3390/diagnostics7040052

**Published:** 2017-10-17

**Authors:** Chiuan Herng Leow, Katja Fischer, Chiuan Yee Leow, Qin Cheng, Candy Chuah, James McCarthy

**Affiliations:** 1 Institute for Research in Molecular Medicine, Universiti Sains Malaysia, Penang 11800, Malaysia; 2 Bacterial Pathogenesis and Scabies Laboratory, QIMR Berghofer Medical Research Institute, Brisbane 4006, Australia; katja.fischer@qimrberghofer.edu.au; 3 Institute for Research in Molecular Medicine, Universiti Sains Malaysia, Kelantan 16150, Malaysia; yee.leow@usm.my; 4 Department of Drug Resistance and Diagnostics, Australian Army Malaria Institute, Brisbane 4051, Australia; qin.cheng@defence.gov.au; 5 Department of Medical Microbiology & Parasitology, School of Medical Sciences, Universiti Sains Malaysia, Kelantan 16150, Malaysia; chuahcandy@usm.my; 6 Clinical Tropical Medicine Laboratory, QIMR Berghofer Medical Research Institute, Brisbane 4029, Australia; james.mccarthy@qimrberghofer.edu.au

**Keywords:** antibody, biomarker, camelids V_HH_, diagnostics, shark V_NAR_, single domain antibody (sdAbs)

## Abstract

Biomarkers are defined as indicators of biological processes, pathogenic processes, or pharmacological responses to a therapeutic intervention. Biomarkers have been widely used for early detection, prediction of response after treatment, and for monitoring the progression of diseases. Antibodies represent promising tools for recognition of biomarkers, and are widely deployed as analytical tools in clinical settings. For immunodiagnostics, antibodies are now exploited as binders for antigens of interest across a range of platforms. More recently, the discovery of antibody surface display and combinatorial chemistry techniques has allowed the exploration of new binders from a range of animals, for instance variable domains of new antigen receptors (V_NAR_) from shark and variable heavy chain domains (V_HH_) or nanobodies from camelids. These single domain antibodies (sdAbs) have some advantages over conventional murine immunoglobulin owing to the lack of a light chain, making them the smallest natural biomarker binders thus far identified. In this review, we will discuss several biomarkers used as a means to validate diseases progress. The potential functionality of modern singe domain antigen binders derived from phylogenetically early animals as new biomarker detectors for current diagnostic and research platforms development will be described.

## 1. Introduction

Early and accurate diagnosis of disease is important for providing appropriate treatment to individuals with most human diseases. Clinical diagnosis remains a mainstay method for clinical care in many settings, such as among febrile patients in endemic areas [1]. However, the overlap of clinical symptoms of many diseases makes misdiagnosis likely and frequent, thereby impeding treatment decisions and epidemiologic information [2].

Microbiological methods represent definitive diagnostic method for various infectious diseases in laboratory settings [3]. Samples tested are predominantly blood, serum, stool and urine that can be collected from both outpatients and hospitalized individuals. Despite high specificity, the requirement of maintaining specific temperature during transportation of clinical specimens to laboratory for processing is problematic, especially in less well-resourced settings [4,5]. Microscopy, when possible, is one of the least expensive methods for laboratory diagnosis. During the Malaria Eradication era, slide-based diagnosis assisted the successful elimination of malaria in many countries [6]. However, obstacles such as time consuming nature of this test, the need for highly trained and experienced staff, and system maintenance have limited the utility of this method for diagnosis of malaria [7,8].

Biomarkers are defined as indicators of biological processes, pathogenic processes, or pharmacological responses to a therapeutic intervention [9]. These include the presence of specific antigens, changes in enzyme activities, and unique changes in DNA and proteins, for example, carcinoembryonic antigen (CEA) in colorectal cancer [10], chitinase enzyme activity in Alzheimer’s disease [11], and circulating tumor DNA in breast cancer [12]. Identification of unique biomarkers of diseases is important for early screening, diagnosis, and monitoring of disease progression. The introduction of high-throughput screening methods, particularly through to genomics, transcriptomics and proteomics, has expanded the knowledge base and potential for diagnosis using biomarkers [13,14]. Methods of directly detecting DNA and RNA have enhanced the precision with which biomarkers of diseases are identified [15]. However, unlike nucleic acid detection methods, post-translation modifications of proteins can complicate the detection of clinically-relevant biomarkers [16].

Antibodies are the primary tool used as recognition reagents to detect biomarkers, and are widely used as analytical tools in clinical settings. For immunodiagnostics, antibodies are now exploited as binders for antigen of interest in a range of platforms, including the enzyme-linked immunosorbant assay (ELISA), immunoblotting (Western Blotting), immunocytochemistry, and the immunoprecipitation assay [17,18]. With the help of sophisticated instruments, antibodies are also used as tools for protein microarrays, flow cytometric analysis, and immunoaffinity analysis [19,20,21]. Therefore, monoclonal antibodies are widely used as a main source of antibodies in rapid diagnostic tests for detecting various infectious diseases such as malaria, dengue, HIV and so on. Antibodies can be isolated from a range of sources, with plasma and serum being the most common sources in laboratory practice [22]. Furthermore, alternative sources, such as chicken egg yolk, have been used to produce functional antibodies against certain biomarkers [23]. Antibodies have also been used as therapeutic agents and animal models such as mice, rabbits, horses and guinea pig are critical to the development of therapeutic antibodies [24].

Small-molecule antibodies are postulated to have better solubility capability [25,26]. Nowadays, the shrinking of intact immunoglobulin into smaller antibody fragments such as scFv, Fv, Fab and dual or tetra-domain fragments has become possible via molecular engineering approaches (Figure S1) [27,28]. An advantage of small antibody fragments is their ease of genetic manipulation due to their smaller size, ease of expression in bacterial systems, and low lot-to-lot variation, and in scaled-up production [29,30,31]. Moreover, the desired antibody fragment repertoire can also be developed from any animal immunoglobulin with an appropriate set of specific primers [32,33].

The introduction of antibody surface display and combinatorial chemistry techniques has greatly allowed the exploration of new binders from various organisms. More recently, natural single domain antibodies (sdAbs) that functioned well in vivo were discovered from a few ancient vertebrates, including V_NAR_ from shark, and V_HH_ from camelids. These groups of minimized antibodies offer the potential of providing advantages over conventional antibodies such as IgG and IgM derived from higher animals [34,35]. The unusual antibodies derived from these groups of animal have been reported to provide promising specificity and sensitivity for their target antigens [36,37,38]. In addition to possessing the capability of better tissue penetration, the peculiar structure of these sdAbs naturally renders them to have better thermostability, enabling them withstand the harsh environment [39,40].

## 2. Classes of Diagnostic Tests that Target Biomarkers

As described above, conventional diagnostic methods such as microscopy remain problematic in many settings. Thus, there is a significant need for new diagnostic techniques that are capable of displaying high specificity and sensitivity [41]. In recent decades, molecular-based diagnostic tools have become well developed, generating new strategies for diagnosis. Examples include polymerase chain reaction (PCR) [42], loop-mediated isothermal amplification (LAMP) [43], mass spectrometry (MS) [44], fluorescence-activated cell sorting (FACS) [45], and single molecule array (SIMOA) technology [46]. In spite of some advantages related to their extremely good specificity, the implementation of these methods is hampered by specific limitations such as their time-consuming nature, cost, need for electricity supply, and lack of experienced technicians, especially in less well-resourced settings. These issues have been extensively reviewed [47,48].

To address these shortcomings, immune-based assays would be ideal tools for biomarker detection in clinical diagnostic settings [49]. As the gold standard for measuring protein content in human body fluids such as blood, the enzyme-linked immunosorbent assay (ELISA) remains the predominant method for detection of antigens of interest (biomarkers) [50]. Furthermore, the invention of new point-of-care systems, such as rapid diagnostic tests (RDTs), has been a major contributor to clinical case management [51,52] patients undergoing self-monitoring at home; for instance, blood glucose reading for diabetics [53]. Many high-throughput antibody-based screening methods have been integrated across various diagnostic platforms to enable rapid identification of biomarkers that may aid individual treatment and monitoring of responses to therapy. Current multiplexed immunoassays are based on multi-marker strategies, in which high-affinity capture ligands (antibodies or proteins/peptides) are immobilized in parallel assays [54,55]. Several commercial multiplexed immunoassay platforms are available on this emerging market, including the Luminex bead-based platform [56], Meso Scale Discovery’s Multi Array Technology [57], and protein array platforms from Whatman [58].

### 2.1. Non-Infectious Diseases and Non-Diseases

Non-infectious diseases, commonly known as non-communicable diseases (NCDs), are the leading cause of morbidity and mortality worldwide. According to the WHO, about 40 million people suffering from cancers, cardiovascular diseases and stroke, chronic respiratory diseases, and diabetes are killed each year in the world [59]. Unlike infectious diseases, NCDs affect people from across the whole world. Of chronic diseases, cancers have been the highest cause of mortality, followed by diabetes and cardiovascular disease [60,61]. In addition, other conditions, such as pregnancy and envenomation, are common cases. Prompt screening for these could render a proper plan, and also the selection of appropriate anti-sera for those who are envenomated. Hence, the development of simple, cost-effective, and low-complexity point-of-care (POC) devices represents an important goal in global health.

#### 2.1.1. Diagnostics for Cancer

Despite substantial investments and progress made in therapy, cancer remains as a major threat to human life across the globe. In the past 5 years, more than 12 million new cases and 7.6 million deaths have been caused by various cancers [62]. The incidence of cancer is projected to continuously increase to 26.8 million new cases and 17.1 million deaths annually by the year 2030 [63]. The most common cancers are prostate, lung, breast, colorectal, liver, stomach and cervical cancers, with lung cancer and breast cancer resulting in the highest mortality among men and women, respectively. The success rate of therapy for cancers can be significantly improved if they are diagnosed early [64].

Antibodies to target biomarkers are widely used, for detecting prostate specific antigen (PSA), specifically expressed in prostate cancer [65], carcinogenic-embryonic antigen (CEA) in colorectal cancer [66], CA15-3 antigen and her-2/neu are proteins associated with breast cancer [67,68], CA19-9 in gastrointestinal cancer [69], while CA125 is a biomarker for ovarian cancer diagnosis [70]. ELISA tests are the current gold standard for identifying cancer biomarkers due to their high sensitivity, specificity, and ability to quantify target antigens [71,72]. The monoclonal antibody (mAb) within NMP22^®^ BladderChek^®^ Test (Inverness Medical Innovations Inc., North America) for example, is an FDA-approved lateral flow immunochromatographic test designed to detect nuclear matrix proteins (NMPs) for bladder cancer. The sensitivity and specificity of this test has been reported to be up to 95% using just 4 drops of urine, with results available in 30 min [73].

Another approach to cancer biomarker detection is by coupling antibodies to electrode-based devices [74]. These systems enable sample profiling on a large scale [75]. An automated diagnostic device with high sensitivity and specificity was developed by Kashani-Sabet et al. to distinguish benign nevi from melanoma via integration of gene expression data and tissue array profiling [76]. Using such tissue proteomics approaches, several potential biomarkers have been identified from melanoma, such as actin-related protein 2/3 complex, subunit 2 (ARPC2), fibronectin 1 (FN1), and regulator of G protein signaling 1 (RGS1) [76].

#### 2.1.2. Diagnostics for Pregnancy

Diagnosis of pregnancy using test kits is one of the most successful immunoassays. Today, many different brands are commercially available worldwide [77,78,79]. In the past, several pregnancy-specific materials were targeted as candidate biomarkers. These include Schwangerschafts protein 1 (SP1) [80], placental protein 14 (PP14) [81] and early pregnancy factor (EPF) [82,83]. In recent years, however, the hormone human chorionic gonadotropin (hCG) has become the most widely detected biomarker for early detection of pregnancy due to its abundance and it being amenable to immunoassay design [84,85,86]. β-hCG is a dimeric glycoprotein with a size of 46 kDa that is synthesized by the trophoblastic tissue in placenta [87], with the amount increasingly secreted after implantation [88]. The first urine pregnancy test using monoclonal antibodies to detect β-hCG based on simple immunological principles was introduced over 30 years ago [89]. Owing to their cost effectiveness, and convenience, pregnancy diagnostic test kits are very widely used by both the general public and healthcare professionals for early detection of pregnancy [90,91].

Assays using traditional polyclonal antibodies have suffered from the drawback of insufficient specificity [92]. Thus, monoclonal antibody (mAb)-based pregnancy tests predominate, and offer nearly 100% sensitivity and specificity for detection of β-hCG at a threshold concentration of 25 mili-IU/mL in urine [93,94]. For example, the Clearview hCG (Unipath, UK) test is able to detect pregnancy on the day of a missed period with just three drops of urine loading to the sample window, within five minutes with accuracy greater than 99% [95]. Most pregnancy test kits presently are very robust because membranes and antibodies are engineered to be protected by a plastic housing, and are sealed to prevent damage from humidity. In addition, when stored at room temperature, the shelf-life for all pregnancy test kits can be up to 2 to 3 years [96].

#### 2.1.3. Diagnostics for Envenomation

Venoms are biological toxins secreted by certain kinds of poisonous animals, such as snakes, scorpions, spiders, hornets and wasps, which usually use them for attacking their prey by targeting the victim’s lymphatic system through biting or “injection” using a special “weapon” or so-called sting [97,98,99]. Snakebite envenomation remains a life-threatening medical emergency worldwide [100]. To prevent inappropriate treatment, which can result in allergy, paralysis or even death of the victims, the establishment of rapid, reliable and specific detection of envenomation is necessary. A diagnostic test is therefore essential to improving emergency management of envenomation to facilitate the provision of appropriate antivenom therapy [101].

Techniques for detection of different snake venoms have been extensively reviewed. Antigen capturing ELISA has been claimed to be the best method for detection of snake venom [102,103]. The first commercial diagnostic kit for detection of snake venoms was introduced by the Commonwealth Serum Laboratories (CSL) in Australia in 1991 [104]. This kit was specially designed to detect the venoms of the five most dangerous snakes in Australia and Papua New Guinea, including Australian Tiger snake, Brown snake, Black snake, Death adder and Taipan [105,106]. The CSL snake venom diagnostic kit (SVDK) promises to provide a rapid, easy-to-use, low-cost and long-shelf-life test. As snake venoms are complex mixtures, a polyclonal antibody is used in each well in SVDK to distinguish particular types of snake venom [107]. The SVDK has been widely evaluated in both humans and animals in many clinical studies, and has shown high sensitivity and specificity [107,108,109]. This kit has been reported to be able to detect 2.5 ng/mL venom in less than 20 min, and has a specificity of 100%. Due to high stability of immunoreagents and low cross reactivity, these characteristics have made SVDK widely used by health workers in tropical countries [107].

Several alternative diagnostic kits have also been developed to identify the venoms of the four common snakes from Asia [110,111,112]. Unlike SVDK, whole blood can directly be used as sample in the AB-microELISA kit. The sensitivity of this assay for detection of venom has been shown to be 10 ng/mL. In addition, 600 μL of whole blood is sufficient for this kit, and results are available within 30 min [113]. Apart from whole blood, serum, urine blister fluids and bite site swabs can also be used as assay samples. However, further studies are ongoing to validate the prototype of AB-microELISA kit for field use [110].

### 2.2. Infectious Diseases

Despite decades of advances, infectious diseases continue to represent leading causes of morbidity and mortality throughout the world [114,115,116]. Millions of people are living under threat of a diversity of diseases caused by bacteria (e.g., *Staphylococcus aureus* and *Salmonella typhi*), viruses (Human Immunodefiency Virus and Hepatitis C virus), fungi (*Candidasis*, *Coccidioides* and *Pneumocystis)* and parasites (malaria and helminths). Infections lead to disability, death and social and economic disruption [117,118]. According to WHO statistics, approximately 15 million people are killed by such diseases in developing countries annually [119]. Moreover, sexually transmitted infections such as syphilis [120], and tropical parasitic infections such as schitosomiasis also cause enormous morbidity [121]. Due to the diversity of environmental conditions in varying developing countries, a need has arisen for the development of simple, accurate, and stable diagnostic tools. The deployment of highly sensitive and specific diagnostic tests is also needed to counteract the spreading of drug resistance of infectious diseases [122].

#### 2.2.1. Diagnostics for Viral Disease

Since the first case of AIDS was reported in early 1980s, HIV/AIDS has caused 1.7 million HIV-related deaths in 2011 alone and estimated about 34 million people were living with HIV worldwide in 2010 [123]. AIDS, referring to acquired immunodeficiency syndrome, is a dangerous infectious disease that eventually causes death without treatment. The etiological agent of AIDS is known as human immunodeficiency virus (HIV), a retrovirus with a single-stranded RNA (ssRNA) [124]. However, many persons who are infected with HIV are not aware of the infection until late in the course of disease.

To increase access to early treatment and prevention, rapid HIV tests play an integral role in HIV prevention activities in both clinical and non-clinical settings [125,126]. ELISA assays are extensively used to screen for the appearance of specific antibodies. To perform a typical indirect assay, the serum collected from a patient is incubated to detect a response to an HIV target antigen; for example, p24, gp24 or gp120. A positive antibody response is then detected by an enzyme-labeled anti-human antibody or an enzyme-labeled antigen [127,128].

Instead of using blood or serum as a sample, HIV infection can also be diagnosed by detecting the presence of anti-HIV antibodies in a patient’s saliva or urine samples [129,130]. For example, the OraSure^®^ assay is a specific salivary test that is designed to determine anti-HIV IgG antibodies from saliva [131]. However, HIV diagnostic tests based on urine and saliva are still not comparable to blood samples in terms of detailed information and the specific characteristics of the HIV subtype responsible for the infection [132].

With the aid of rapid tests, screening tests can be conducted in hard-to-reach patient populations. Unlike high-throughput EIA screening tests, RDTs can perform well for HIV diagnosis even when the volume of samples is low [133]. With the administration of combination anti-retroviral therapies, the transmission rate of HIV can be reduced from over 25% to less than 2% for those infected pregnant patients [134]. Recently, the US FDA has approved four types rapid tests for screening HIV-1 infection, including Murex^®^ Single Use Diagnostic System HIV-1 Test (Murex Diagnostics, Inc., USA), OralQuick^®^ Rapid HIV-1 Antibody Test (OraSure Technologies, Inc., USA), Reveal^®^ Rapid HIV-1 Antibody Test (MedMira Laboratories Inc., Canada), and UniGold Recombigen^®^ HIV (Trinity Biotech PLC, Ireland) [135].

However, false-negative results can occur in individuals who are in the acute phase of infection [136]. Diagnostic tests that directly detect the HIV p24 antigen in serum samples have been shown to be superior to antibody-based detection tests for early infection [137]. For instance, the commercial Vidas Duo assay (bioMerieux Inc., March-L’Etoile) is a fourth-generation ELISA. This assay possesses the advantage by decreasing the diagnostic window to an average of 7 days. In addition to detecting anti-HIV antibodies, this kit targets the HIV p24 antigen that is present in the blood of an HIV infected individual in the early phase [138].

#### 2.2.2. Diagnostics for Bacterial Disease

Typhoid fever is a serious systemic illness caused by the *Salmonella enterica* serotype Typhi. This disease represents the most common cause of community-acquired bacteremia in developing countries [139]. The annual global incidence of typhoid fever is over 21.5 million cases, resulting in more than 200,000 deaths [140]. The emergence of multidrug resistance *S. typhi* has complicated treatment [141]. Therefore, rapid and accurate diagnosis is essential to provide early antimicrobial treatment, for preventions of mortality cases, and for the control of disease transmission [142].

In the past, the Widal test was the most widely used serologic test for detection of host antibodies. This agglutination test targeted typhoid antisera such as lipopolysaccharide (O) and flagellar (H), and Vi antigens of *S. typhi* [143,144]. However, tests was limited by false-positive results due to cross-reaction of the antigenic determinants with non-typhoid *Salmonella* or other tropical diseases such as malaria and dengue [145,146]. False-negative reactions may also occur if the blood sample is collected too early from the infected individuals [147]. The Widal test is not a satisfactory test for diagnosing typhoid fever in endemic areas [148]. Recently, a new generation of rapid serologic tests has been developed, for example Linear Cromotest^®^ (Linear Chemicals, Barcelona, Spain). This test aims to detect host IgM and IgG antibodies which are specific to O and H antigens of *S. typhi*. The highest reported specificity (50%) and sensitivity (95.2%) limited the accuracy of diagnosis of typhoid fever at two sub-Saharan African sites [149].

TUBEX^®^ (IDL Biotech AB, Bromma, Sweden) is semiquantitative colorometric rapid test that use polystyrene particle agglutination to detect anti-O9 IgM antibodies specific for group D of *S. typhi*. This kit enables rapid diagnosis of typhoid fever patients, with only 3 min per test at room temperature [149]. The TUBEX^®^ test kit is designed for detection of antibodies in the patient’s serum by inhibiting the binding between an indicator antibody-bound particle and a magnetic antigen-bound particle [150]. Thus, an acute stage of *S. typhi* infections can be indicated by elevated levels of anti-O9 IgM antibodies in combination with typical clinical symptoms of typhoid fever [151,152]. However, the TUBEX^®^ colorimetric reaction may be subject to false-positive results due to hemolyzed samples in individuals with recent *S. enteritidis* infection [150]. Despite the promising test performance, the requirement for additional laboratory equipment has limited this test kit being used in resource-limited endemic regions [149].

Another rapid test for diagnosing *S. typhi* of typhoid fever is Typhidot^®^ (Malaysian Biodiagnostic Research, Bangi, Malaysia). This is a dot EIA test that detects either host IgM or IgG antibodies against the *S. typhi* antigen [153]. The target antigen used in the assay is neither an O nor H antigen, but a 50 kDa outer membrane protein (OMP) antigen of *S. typhi*. This recombinant protein is coated on a nitrocellulose strip for detection of the antigen-antibody complex by employing an anti-human antibody conjugated peroxidise and a chromogenic substrate [154]. Due to host immune response, the 50 kDa OMP is a good antigen to identify *S. typhi* specific antibodies in the sera of individuals with typhoid [155,156]. Since the IgG antibody can persist in the host for more than 2 years, the detection of IgG antibodies can lead to false positive results by confusing between acute or convalescent cases [157]. An upgraded version of Typhidot-M^®^ has been introduced. It activates antibodies to allow accessibility of OMP antigens to the specific IgM. With this approach, detection of acute typhoid infection can be obtained within 3 h [158]. Both Typhidot^®^ and Typhidot-M^®^ are simple, fast, specific, sensitive, and economical dot diagnostic assays for providing early detection of *S. typhi* infections. Evaluation studies on the Typhidot^®^ and Typhidot-M^®^ tests in clinical settings showed that these tests performed better than the Widal test and conventional gold standard culture methods [158]. Both Typhidot^®^ and TUBEX^®^ kit have reportedly given good performance for diagnosis of typhoid fever in small cohorts of hospitalized patients [159,160].

#### 2.2.3. Diagnostics for Parasitic Diseases

Malaria remains a severe parasitic disease leading to high morbidity and mortality in tropics [161]. This protozoan parasitic disease is transmitted by female Anopheles mosquitoes. According to the World Malaria Report 2012, it was estimated that about 219 million cases of malaria, causing 660,000 deaths, occurred throughout the world in 2010. Africa is the most prevalent region, where up to 90% of all malaria deaths occur [161]. Four species of *Plasmodium* parasites are well-known as causative agents for human malaria, namely *P. falciparum*, *P. malariae*, *P. ovale*, and *P. vivax*. However, the simian species *P. knowlesi* has been recently identified as a new species that can cause malaria infection in humans [162]. This mainly occurs in Malaysian Borneo [163] and other South East Asian countries [164,165]. Of these species, *P. falciparum* is the most pathogenic**,** accounting for the majority of febrile illness and death [166]. Therefore, it is crucial to understand the important parameters in the transmission of the disease, and develop effective diagnostic strategies for its prevention and control.

Malaria rapid diagnostic tests (RDTs) using antigen capture technology were developed in the early 1990s, and have led to much-improved access to diagnostic tests for malaria. Such devices are intended to provide simple, swift, accurate and reliable diagnosis of malaria in areas where microscopic diagnosis is not applicable [167,168]. Other advantages of RDTs are that they do not require complex methodologies, intensive training, and electricity supply, thus representing promising diagnostic tools in remote areas [169,170].

A variety of antigens have been investigated as candidate targets for malaria RDTs. *Plasmodium falciparum* histidine rich protein 2 (PfHRP2), *Plasmodium* lactate dehydrogenase (pLDH), and parasite fructose 1,6-biphosphate aldolase (Aldolase) are predominantly used as biomarkers in malaria RDTs. PfHRP2 is water soluble protein that is specific to *P. falciparum*, and is produced by parasite 2 h after invasion of red blood cell [171,172]. The molecular weight of the secreted *Pf*HRP2 varies from 60 to 105 kDa [173]. pLDH is a soluble glycoprotein enzyme produced by the asexual and sexual stages of parasites [174]. Different isomers of pLDH can be identified in all human malaria species [175]. Aldolase is an enzyme of the parasite glycolytic pathway that is also synthesized by all human malaria species [176]. Although three types of tissue-specific aldolase isoenzymes can be found in all higher vertebrates, *P. falciparum* and *P. vivax* possess only one aldolase isoenzyme, which is also similar to that possessed by *Giardia lamblia* and *Trypanosoma brucei* [177].

Nowadays, malaria RDTs have been developed into a range of test formats, including dipstick, strip, card, pad, well, or cassette devices [175]. PfHRP2-detecting tests were the first type of RDT to become available specifically for *P. falciparum* detection (ParaSight-F^®^ and ICT^®^), where mAb against PfHRP2 were used as signal and capture antibodies [178,179]. It was followed soon after by pLDH, and Aldolase detection tests, such as OptiMAL^®^ which are able to detect all four human *Plasmodium* species (pan-malaria) [180,181,182]. Polyclonal antibodies have been used as capture antibody in the qualitative and quantitative immunoassay test for targeting pLDH [183]. Meanwhile, monoclonal antibodies against parasite Aldolase that are pan-specific have been used in a combined “*P. f/*pan” immunochromatographic test to detect non-*P. falciparum* spp., along with PfHRP2 [184]. Recently, the increased demand for RDTs has resulted in more than 200 malaria RDT products from 60 manufacturers currently being available in the global market [185].

## 3. Currently Available Antibody Binders for Detection of Biomarkers

Antibodies can be classified into three different categories: polyclonal antibodies, monoclonal antibodies, and recombinant antibodies [186]. Polyclonal antibodies (Polyclonal Abs) are heterogeneous antibody mixtures that are derived from multiple plasma cell lines. Owing to their complexity, polyclonal antibodies have excellent properties for recognizing complex antigens carrying numerous epitopes [187]. A monoclonal antibody (mAb) is a homogeneous antibody generated from a single B lymphocyte clone. Antibodies produced in mAb format have extremely high specificity against a single epitope on antigens [188]. Recombinant antibodies or antibody fragments (rAbs) are antibodies generated using molecular techniques in laboratory. They are aimed at improving the sensitivity, selectivity, stability and immobilization properties in diagnostic applications, for example, in biosensors [189].

In making the decision to use or generate polyclonal, monoclonal or recombinant antibodies, several factors should be considered, including commercial availability, animals to raise, types of applications, time length and cost [186]. A comparison of parameters for producing different source of antibodies is shown in Table 1.

### 3.1. Monoclonal Antibodies

The first description of mAb production was by Nobel Prize winner Kohler and Milstein in 1975 [190]. The fusion technique between splenic B cells and myeloma cells, termed the hydridoma technique has revolutionized immunology. The production of mAbs is not influenced by the animal sources used, thus, giving mAbs a better homogeneity and consistency in scaled-up production [191]. mAb technology has been widely applied in biomedical research and the pharmaceutical industry.

Unlike polyclonal Abs, the monospecificity of mAb enables targeting a single epitope. This permits a range of applications, including targeting members of a protein family and evaluating changes in molecular conformation and protein-protein interactions. However, the functionality and sensitivity of mAbs can be reduced by small changes in the structure of antigen determining regions, or even by minor changes in pH or salt concentration. One advantage is that mAbs can be produced at greater concentration and much higher purity than polyclonal Abs [191]. Disadvantages of mAb can be overcome using combinations of multiple mAbs specific to desired antigens. However, this pooling method can be difficult, costly, and time consuming [191]. Nowadays, the mass production of mAbs through the ascites method has been largely replaced by in vitro technology such as bioreactors due to the constraint of needing use of mice as host animals [192,193,194,195,196].

### 3.2. Limitations of Conventional Monoclonal Antibodies

**Figure 1:**
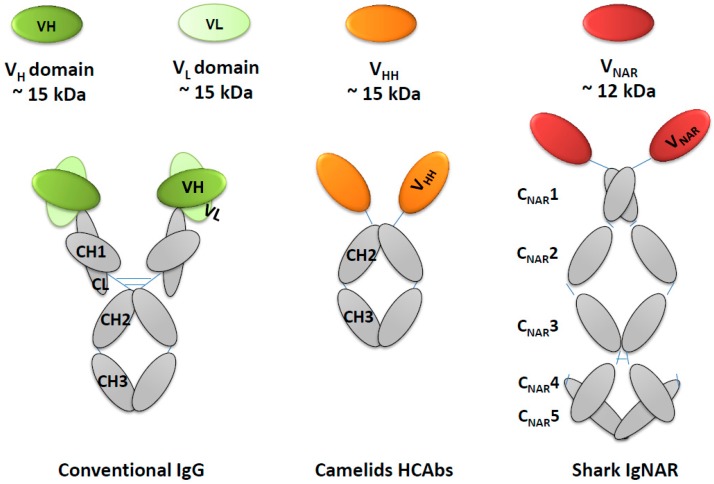
Schematic representation of the comparison conventional antibody IgG with natural single domain antibodies derived from camelids and sharks. Single V (colored ovals); C domains (grey colored). Each color in V domains VH, VL, or single heavy domain represents the specific source of animals.

As bivalent antibodies, IgG represents the most abundant immunoglobulin proteins (approximately 85%) found in all mammalian serum (Figure 1) [197]. Due to their ability to confer high affinity and retention times, monospecific IgGs are the preferred reagents in biomedical research, as well as in therapeutic and diagnostic applications. However, several practical drawbacks are apparent for diagnostic reagents based on conventional IgG antibodies. The complex architecture and large molecular size (~150 kDa) may result in weak bindings, when small sizes or small amounts of protein antigens are not easily recognized by the concave surfaces of CDR loops [198,199].

More importantly, there is a concern in the application of RDTs in tropical countries regarding their shelf life, because some of these antibodies are susceptible to degradation by excessive temperatures (>40 °C), or by storage for extended periods under conditions of high humidity [200]. To overcome humidity, most RDT devices are now protected in a hermetically sealed plastic packet, or a desiccant is used to ensure that the test strips remain dry [201]. However, it is still a challenge to protect RDTs from high ambient temperatures, resulting in reduced performance of RDTs, especially in tropical countries, where temperatures regularly rise to 45 °C. Hence, for most RDT devices a storage temperature between 4 to 30 °C is recommended. However, this condition is difficult to meet, especially in endemic areas where refrigerated storage systems may not be available [202,203].

To address these problems, initial attempts to generate single domain antibody fragments by separating expression of individual VH or VL units was introduced Ward and co-workers [204]. However, this approach reportedly resulted in solubility problems in aqueous solvents, higher cost, a more time-consuming process, and the requirement of sophisticated protein engineering approaches [205]. Moreover, its failure to recognize selected mAbs on conserved epitopes of specific antigens due to unbound reactivities mediated by the Fc region hinder its utility for diagnostic applications [206,207].

With the emergence of DNA engineering, surface display has been widely used to discover new antibody fragments for the purposes of diagnostic and therapeutic application. As a consequence, a range of different types of new antibodies has been investigated, aiming to overcome the limitations presented in the conventional antibodies.

## 4. Phage Display Technology for New Biomarker Binder Discovery

Screening phage display libraries is a powerful tool for identifying specific binders from libraries that contain a large diversity of targets [33,208]. Library construction is achieved by splicing a repertoire of genes (genotype) that encodes the peptide into a gene that encodes a capsid structural protein (phenotype). The “displayed” peptides are included in the capsid layer on the phage surface. Ideally, these proteins should not interfere with the phage structure [209].

Recombinant DNA technology has enabled phage library construction whereby billions of variant peptides and proteins are able to be presented on the surface of the phage [210,211]. From this diversity library, binders specific to proteins of interest with high affinity can be selected by biopanning. This technique facilitates understanding of protein–ligand interactions [212], antigen-antibody interactions [213,214], and permits improvement of the affinity of proteins to their binding partner [215,216]. For instance, phage display antibody libraries with diversities as high as 10^10^ can be established using display technology [217,218]. Recently, surface display technology has expanded to include ribosome display [219], yeast surface display [220], and mammalian cell display [221]. Such technologies have enabled the exploration of new antibodies that may not otherwise have been discovered, from humans and animals including shark, camel, llama, and lamprey [222,223,224,225].

**Figure 2:**
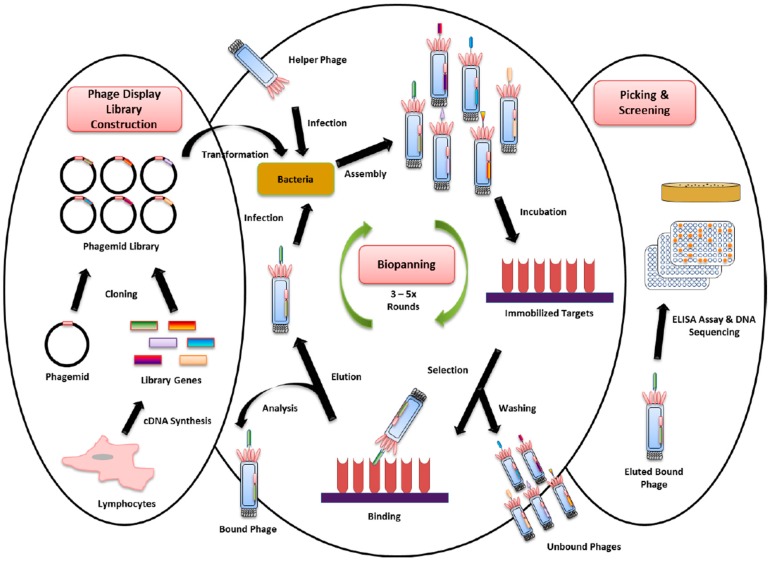
Principle of filamentous bacteriophage M13 phage display using a phagemid vector. Gene encoding for millions of variants of libraries are cloned into a phagemid vector carrying the gene encoding for one of five phage coat proteins (pIII). Large phage libraries can be obtained by transforming *E. coli* with phagemids and rescue of phages with helper phage. Hence, phages displaying the specific-binding antibodies against immobilized targets can be selected and isolated by several rounds of biopanning. These involve binding, washing, elution, infection and amplification. The eluted bound phages are subsequently screened by ELISA assay and followed by DNA sequencing prior to their protein expression and purification.

Antibody phage display libraries have been used extensively for isolation of high-affinity specific binders against unique antigens from different targets [226,227,228,229,230]. Three types of antibody libraries are typically constructed: naïve, synthetic, and immunized libraries [231]. A naïve antibody library refers to the repertoire of antibody genes derived from non-immunized donors. Synthetic antibody libraries are constructed using synthesized V-gene fragments with randomized CDRs, whereas immunized libraries are based on a host immunized with the target antigen of the disease [232]. The principle of the phage display is represented in Figure 2, indicating the workflows of library construction, biopanning, and clone screening prior to protein expression and purification for functional assays.

## 5. Natural Single Domain Antibodies

The evolution of immunoglobulins from invertebrates began ~550 million years ago [233]. With the emergence of antibody surface display technology, interest has increased in new binders from less commonly used animals, including V_HH_ from camelids, and V_NAR_ from sharks. The unusual antibodies derived from these groups of animals have been reported to provide promising specificity and sensitivity for target antigens [36,37,38]. The availability of new binders derived from lower vertebrates is now discussed.

### 5.1. V_HH_ Heavy Chain Domain in Camelids

As in all mammals, members of the camelid family produce immunoglobulin G which comprises two heavy chains and two light chains fused with disulfide bonds. However, unlike the V_H_ and V_L_ domains in conventional antibodies, a unique subclass of immunoglobulin containing only a heavy chain domain and lacking a light chain was found in the circulatory system of camelids. Owing to its peculiar structure, this antibody has been configured as “heavy chain only” antibodies (HCAbs) [234]. The capacity of camelid HCAbs to retain the reversibility and binding activity after heat denaturation has enabled new applications where transient heating may occur [235,236,237].

HCAbs are slightly different from IgG, in that they also include both a constant (Fc) and variable domain. The isolated variable domain region of camelids HCAbs is known as V_HH_ (variable domain of the heavy chain of HCAbs) or Nanobody^®^ (Nb; Ablynx) [238]. Similar to the products of protein engineering the V_H_ domain from other mammals, the N-terminal of V_HH_ is naturally utilized as a binding surface to interact with the target antigen [234]. The molecular weight of V_HH_ is 15 kDa, ten times lower than that of an intact conventional antibody (Figure 1). It was thereby considered the smallest possible antibody fragment, and has attracted the interest of many scientists [239,240,241].

The major advantage of the V_HH_ antibody is its greater solubility compared to classical V_H_ [205]. This is due to the hydrophilic amino acid substitution present in the framework 2 region. Meanwhile, the single coding exon of less than 450 base pairs facilitates genetic engineering of V_HH_ fragments [240,242]. In addition, on account of its smaller antigen binding surface area, the unique CDR3 region enables the heavy domain of camelids to penetrate into antigen cleft regions that are not easily recognized by conventional antibodies [243,244]. From a phylogenetic prospect, it is conceivably possible to produce humanized V_HH_ [245], a process that may be “easier” than the complicated manipulation required to “humanize” murine or other more distant species to reduce an alloresponse, such as the human antimouse antibody (HAMA) response [246]. Furthermore, due to their high intrinsic domain stability, camelid V_HH_ is now under investigation as a probe for diagnostics [247,248]. The diagnostic potential of camelid V_HH_ as a probe in immunodetection systems offers possibilities for improving the diagnosis of infection [249], cancers [250], and caffeine contaminants in the food and beverage industries [251,252].

### 5.2. V_NAR_ Heavy Chain Domain in Sharks

A class of naturally occurring single variable domain antibodies was discovered in the serum of elasmobranch cartilaginous fish during the early 1990s [253,254,255]. These natural functional repertoires were termed immunoglobulin new antigen receptors (IgNARs). IgNARs are an unconventional and unique class of proteins found in sharks, including nurse sharks (*Ginglymostoma cirratum*) [256], wobbegong sharks (*Orectolobus maculatus*) [257], smooth dogfish (*Mustelus canis*) [258], banded hound sharks (*Triakis scyllium*) [259], and horn sharks (*Heterodontus francisci*) [260]. Investigations has revealed that IgNARs function as antibody and immune response mediators in sharks. However, unlike camelid V_HH_ domains, the IgNAR V region is more similar to light chain and T-cell receptor variable regions than to other VH regions [254,261].

Several desirable biological properties of IgNAR V-domains have been identified, and their potential as alternative antigen binders explored [257,258,262]. The natural habitat of sharks has resulted in them evolving an extraordinarily stable immune system such that the functionality of antibodies can be retained in a harsh environment [263]. Electron microscopic studies have indicated that the intact IgNAR exists as a disulfide-bonded homodimer that consists of a polyprotein with one variable domain (V_NAR_) and five constant domains (C_NAR_) (Figure 1) [264].

Similar to the camelid V_HH_, the V_NAR_ has only a heavy chain domain. However, the cross-species conservation of amino acid sequences with human VH is extremely low in V_NAR_ (~25%), whereas it is more than 80% homologous to classical V_H_ scaffolds in camelid V_HH_ [255,265]. It is hypothesized that IgNARs lack many residues that exist in conventional antibodies; these are replaced by other hydrophilic residues. The greatly truncated CDR2 region, herein defined as an HV2 region, has created a signature hallmark for shark V_NAR_. Due to this unusual structure, the single variable heavy domain proteins of shark IgNARs are currently the smallest antibody fragments observed in the animal kingdom, having a size of only 12 kDa. Yet, in combination with the peculiar feature of a long CDR3 region, these V_NAR_ domains thought to more readily penetrate cleft regions of antigens, thereby increasing the opportunity to target small target epitopes that may not be accessible to conventional IgG [266].

In terms of heat-stability, V_NAR_ also possesses refolding properties as found in camelid V_HH_. The ability to retain fully functional binding-specific activity after exposure to temperatures of up to 95 °C may make V_NAR_ ideally suited to protein array and diagnostic applications where transient heating may occur as part of the protein immobilization process [258,267]. It is partly due to the presence of cysteine residues in these single domain antibodies, making an extraordinary structure conformation [268].

V_NAR_ domains are more easily produced as recombinant proteins than conventional antibodies. Additionally, due to hydrophilic residues present within V_NAR_ surfaces, high yields of expressed proteins associated with high solubility are achievable, and are thus easy to express in prokaryotic systems [257]. Therefore, the potential utility of V_NAR_ as an alternative binder for clinical applications is now being investigated in a variety of research areas for diagnostic and therapeutic purposes.

## 6. Use of Different Binders for Specific Applications

To date, mammals remain the main source of intact antibodies for targeting diseases. However, with the aid of DNA technology, a number of new antibodies have been engineered as smaller single domain fragments to improve of immunoassays, immunosensors, and imaging probes in various applications. As described recently, the discovery of natural single heavy domain antibodies from camelids V_HH_, shark V_NAR_, and lamprey VLRs offer some advantages over conventional antibody fragments. This range of natural antibodies is expected to open applications such as enzyme inhibitors and intrabodies, and as detection units in biosensors or immunodiagnostics. In the following section, the deployment of different binders in specific diagnostic applications will be reviewed.

### 6.1. Applications of Camelids V_HH_ Domain or Nanobodies^®^

To monitor infections, single domain antibodies naturally derived from camelids (nanobodies) may enable superior detection of species-specific antigens to classical monoclonal antibodies in immunodiagnostic tests. Trypanosome infection causes African sleeping sickness and Chagas disease. Both are severe parasitic diseases caused by protozoa of the genus *Trypanosoma*. Sleeping sickness disease is mainly reported in rural Africa. The antigenic variation strategy adopted by this parasite represents a major barrier to the immune system to eliminate it. Consequently, it is difficult for specific mAbs to detect genus-specific antigens [269]. By adopting an in vitro selection method, novel nanobody clones that showed specificity to *T. evansi* at a species level and genus-specific reactivity against various *Trypanosoma* species were isolated. Due to their small sizes, nanobodies were shown to be capable of penetrating into the conserved epitopes of antigens that are inaccessible to classical mAbs [270].

Cysticercosis is a serious tissue infection caused by larval cysts of the pork tapeworm that is prevalent in many low-income countries [271]. Monoclonal antibodies that are currently deployed in sandwich ELISAs are mainly genus-specific against *Taenia* sp., but poorly specific at a species level to identify *Taenia solium*, the major *Taenia* species threatening human health [272,273]. To circumvent such limitations, an in vitro selection of nanobodies from immunized dromedaries was developed to recognize a specific marker on *T. solium*. After in vitro selection, the nanobodies showed no cross-reactivity against other livestock *Taenia* species, while having a very specific response to a specific 14 kDa glycoprotein (Ts14) in *T. solium*. Therefore, nanobodies showed potential as an alternative to genus-species mAb for developing unambiguous ELISA tests for human cysticerosis [249]. Apart from being used as diagnostic reagents for infectious diseases, nanobodies have been identified as alternative binders to analyze the compositions of substances in the food and beverage industries. Due to their excellent thermal stability, nanobodies showed superior performance to classical mouse mAbs in ELISA at measuring caffeine concentration in hot and cold beverages [252].

Camelid sdAbs have recently been applied in ELISA methods to detect a wide range of small molecules, including explosive materials (trinitroluene or TNT) [274], agents of bioterrorism (Botulinum A neurotoxin) [235], toxins (ricin, cholera, staphylococcal enterotoxin B) [275], scorpion toxin [276], and viruses (HIV, rotavirus, Vaccinia, and Marburg) [277,278,279]. Owing to the combination of several favorable properties, camelid nanobodies have also been employed in some sophisticated devices to diagnose diseases. In miniature device development, the advanced features of highly stable and unique conformational structures of nanobodies have permitted overcoming many problems faced by traditional whole antibodies and scFv fragments such as cross-reactivity and nanoparticle agglutination. The development of biosensors coupled with nanobodies (nanoconjugates system) has enabled significant improvement in the performance of a device at identifying harmful bacteria (*Staphylococcus aureus*) at down to a nanometer scale within 10 min [280].

Nevertheless, mAbs remain common binding agents for identifying and tracing tumor-associated proteins for noninvasive in vivo imaging. However, due to their limitations, particularly their large size (150 kDa) and their Fc regions, mAbs penetrate poorly into solid tumors [281]. The emergence of native nanobodies offers the possibility of resolving such problems, and thereby promises the development of probes for diagnosing tumor markers such as EGF receptors [282]. This will enable cancer staging predictions in the blood circulation such as prostate-specific antigen [283]. In view of therapeutic potential, Argen-X (www.argenx.com) has recently developed SIMPLE Antibody^TM^ (chimeric humanized IgGs) that derived from llama VH and VL domains for the treatment of severe autoimmune diseases and cancer. More applications using camelids V_HH_ targeting specified antigens from various diseases is summarized in Table 2.

### 6.2. Applications of Shark V_NAR_ Domain

Evidence that IgNAR is part of the shark adaptive immune response was demonstrated in work where increasing levels of hen egg lysozyme (HEL) specific IgNAR developed in shark sera after 4–5 months immunization [224]. The peculiar structure of the shark IgNAR variable domain renders it amenable to the creation of synthetic peptide mimetics to target specific epitopes that are inaccessible to conventional antibodies [264]. Therefore, V_NAR_ may be suitable as new molecular reagents for research and diagnostic applications, and for immunotherapeutic applications.

Apical membrane antigen-1 (AMA1) is a highly polymorphic 83 kDa merozoite surface protein that is essential for erythrocyte invasion by malaria parasites [312]. A V_NAR_ isolated from a wobbegong shark showed high binding affinity to *P. falciparum* AMA1 through its unique CDR3 region after undergoing affinity maturation [313]. The binding specificity of a monovalent V_NAR_ clone to *P. falciparum* AMA1 was comparable with commercially available binding reagents, derived from conventional polyclonal sera, monoclonal antibodies, small fragments (Fab, scFv) and peptides [314]. Foley and co-workers demonstrated that the heat stability of purified recombinant V_NAR_ was superior to that of conventional mAbs by targeting immobilized *P. falciparum* AMA1 in various format at 45 °C, and the refolding property of V_NAR_ was retained when the temperature increased to 80 °C. The excellent stability property at extreme pH and resistance to proteolytic cleavage was further evidenced by incubating V_NAR_ with homogenized murine stomach tissues under in vivo conditions [267]. Based on these properties, it was proposed that V_NAR_ domains have potential for development as alternate binders for malaria diagnostics platforms.

Human periodontal disease is an advanced gingivitis caused by the bacterial pathogen *Porhyromonas gingivalis* [315]. Late treatment often leads to dental loss due to the accumulation of lysine gingipain (KgP). KgP is a high molecular weight polyprotease produced by *P. gingivalis* [316]. This bacterial toxin is responsible for destruction of dental tissue of host by suppressing the secretion of specific lytic enzymes from immune system [317]. Nuttall and co-workers (2002) identified two distinct clones specific to KgP from a wobbegong shark V_NAR_ phage display library. The high stability and binding affinity towards *P. gingivalis* KgP indicated the potential for V_NAR_ as a valuable source of single domain binding reagents [318].

In recent studies, shark V_NAR_ domains have been reported to detect markers from viral diseases at a greater sensitivity. Ebolavirus hemorrhagic fever (EVHF) is a highly lethal disease caused by Bundibugyo virus (BDBV), Sudan virus (SUDV), Tai Forest virus (TAFV), and Zaire Ebolavirus (ZEBOV) [319,320,321]. Shark V_NAR_ and murine scFv phage display libraries have been generated against specified markers on Zaire Ebolavirus. The results indicated that the sensitivity and thermal stability of shark V_NAR_ against viral nucleoprotein (NP) was superior to murine mAb and scFv in this class [262].

As in the case with camelids nanobodies, highly diversified shark V_NAR_ libraries have also been used to detect different kind of toxins, including staphylococcal enterotoxin B (SEB), ricin, botulinum toxin A (BoNT/A) complex toxoid [322], and cholera toxin (CT) [258]. In addition to identifying markers from non-infectious diseases, the intrabody of V_NAR_ has been reported to recognize immunosilent targets in humans, for example the 70 kDa translocase of outer membrane (Tom70) [323]. Owing to the findings of negligible cross-reactivity with other unspecified antigens, and superior heat stability, shark V_NAR_ domains may be potent source of thermal sdAbs over conventional antibodies in diagnostic and biotherapeutic applications. The applications of recombinant shark V_NAR_ against specified antigens from various diseases is summarized in Table 3.

## 7. Conclusions

Diagnosis by biomarker detection has become a new trend in a wide range of diagnostics, as it could be beneficial for personalized therapy. Conventional antibodies such as IgG and IgM derived from mammals are commonly used as antigen binders in immunoassays for identification of particular disease. With the emergence of genetic engineering, the production of monoclonal antibodies is undoubtedly overcoming many shortcomings presented in polyclonal sera. In order to achieve greater binding efficacy, molecular scientists have continually explored new binders with smaller size and better durability. Natural small-molecule single domain antibodies (sdAbs), functional but not structurally related to the conventional antibodies, have recently been discovered in some ancient animals. They are known as V_HH_ or Nanobodies^®^ from camelids, V_NAR_ from sharks, and, recently, variable-like lymphocytes (VLRs) from lamprey fish. Unlike mammal antibodies, these sdAbs are only composed of heavy protein chains, thereby making them the smallest antibodies thus far. Although most remain at the stage of proof of concept, the advantages of natural sdAbs including better solubility, tissue penetration, stability towards heat and enzymes, and comparatively low production costs offer the posibility of advances in finding new binders for use in research, diagnostic and clinical.

## Figures and Tables

**Table 1:** Comparison of parameters for producing monoclonal, polyclonal, and recombinant antibodies.

Characteristics	Monoclonal Antibody	Polyclonal Antibodies	Recombinant Antibodies
Cost to produce	+++	+	++
Difficulty of production	+++	+	++
Skills or training	+++	+	++
Time scale	+++	+	++
Specificity and affinity	+++	+	+++
Amounts	+	++	+++
Commercial availability	++	+++	+
Variability	+	+++	+

“+” represents the least, “++” represents the moderate, and “+++” represents the most (Adapted from [186] with permission from publishing journal).

**Table 2:** The applications of camelids V_HH_ against specified antigens from various diseases.

Target Antigens	Diseases	Applications	Reference
HER2	Breast cancer	Diagnostic	[284,285,286]
TNT	Explosive	Diagnostic	[274,287]
Ts14 glycoprotein	*T. solium* cysticercosis	Diagnostic	[249]
LMM, ES, CSE, TSB, LLGPs, VF of *T. solium*	Neurocysticercosis	Immunodiagnosis	[249]
VEGF-A_165_	Neoangiogenesis	Diagnostic and therapeutic	[288]
HPV-16 L1 protein	Cervical cancer	Diagnostic and therapeutic	[289,290]
DARC	Malaria (by *P. vivax*)	Diagnostic or therapeutic	[291]
Poliovirus type 1 Sabin strain particles	Poliomyelitis	Diagnostic and therapeutic	[292,293]
CD105	Angiogenesis related tumors	Diagnostic and therapeutic	[294,295]
HSP-60	Brucellosis (Livestock)	Diagnostic and vaccine	[222,296]
Caffeine carboxylate KLH	Beverages	Detection and separation	[251,252]
SEB	Toxin	Sensor and diagnostic	[275]
Ricin	Toxin	Sensor and diagnostic	[275]
BoNT/A	Toxin	Sensor and diagnostic	[235,297,298]
CEA	Colon cancer	In vivo imaging	[299,300,301]
VCAM1	Atherosclerotic plaques	Molecular imaging	[302,303,304]
EGFR	Tumours	Detection and imaging	[305,306]
Scorpion AahII	Toxin	Neutralizing and therapeutic	[276,307]
Heat-killed *B. melitensis* Riv1	Brucellosis	Diagnostic, therapeutic and vaccination	[282,308,309,310]
RSV protein F	Acute lower respiratory tract	Therapeutic	[311]
vWF	Thrombosis	Therapeutic	www.ablynx.com
TNFα, IL-6R, IgE	Rheumatoid arthritis	Therapeutic	www.ablynx.com
RANKL	Bone metastasis	Therapeutic	www.ablynx.com
RSV	bronchiolitis and pneumonia	Therapeutic	www.ablynx.com
DR5	Solid tumors	Therapeutic	www.ablynx.com
Not stated	Alzheimer’s disease	Therapeutic	www.ablynx.com

**Table 3:** The applications of shark V_NAR_ against specified antigens from various diseases.

Target Antigens	Diseases	Applications	Reference
AMA1 (*P. falciparum*)	Malaria	Diagnostic	[313,314]
Zaire ebolavirus viral nucleoprotein	Ebolavirus Haemorrhagic Fever	Diagnostic	[262]
Cholera toxin	Toxin	Diagnostic	[258]
Tom70	Human immunosilent target processes	Detection	[323]
BoNT/A	Toxin	Sensor and diagnostic	[322]
Ricin	Toxin	Sensor and diagnostic	[322]
SEB	Toxin	Sensor and diagnostic	[322]
HBeAg	Hepatitis B virus	Immunolocalization and diagnostic	[324]
Kgp protease (*P. gingivalis*)	Periodontal disease	Neutralization	[318]
Nonfibrillar oligomer formation	Alzheimer’s disease	Modelling	[325]
rhTNFα	pro-inflammatory cytokine	Therapeutic	[260,326]
mAb idiotope	Cancer	Therapeutic	[327]
GPCR’s ion channels		Therapeutic	www.adalta.com.au
Anti-thrombotic drug targets	Cardiovacular disease	Diagnostic and therapeutic	www.adalta.com.au
Idiopathic pulmonary fibrosis	Inflammation	Therapeutic	www.adalta.com.au
Multiple sclerosis	Central neuron system disease	Therapeutic	www.ossianix.com
Botulinum toxin light chain protease	Gastrointestinal tract	Therapeutic	www.ossianix.com
Myostatin	Neurological disease	Therapeutic	www.ossianix.com
Uveitis	Eye inflammatory	Therapeutic	www.elasmogen.com

## Supplementary Materials

The following are available online at www.mdpi.com/2075-4418/07/4/52/s1.
