# Supplementary material for: Single Domain Antibodies as New Biomarker Detectors
Source: Diagnostics (Basel). 2017 Oct 17;7(4):52. doi: 10.3390/diagnostics7040052 (PMC5745390; doi:10.3390/diagnostics7040052)
Supplement: Supplementary file 1 [file diagnostics-07-00052-s001.pdf]

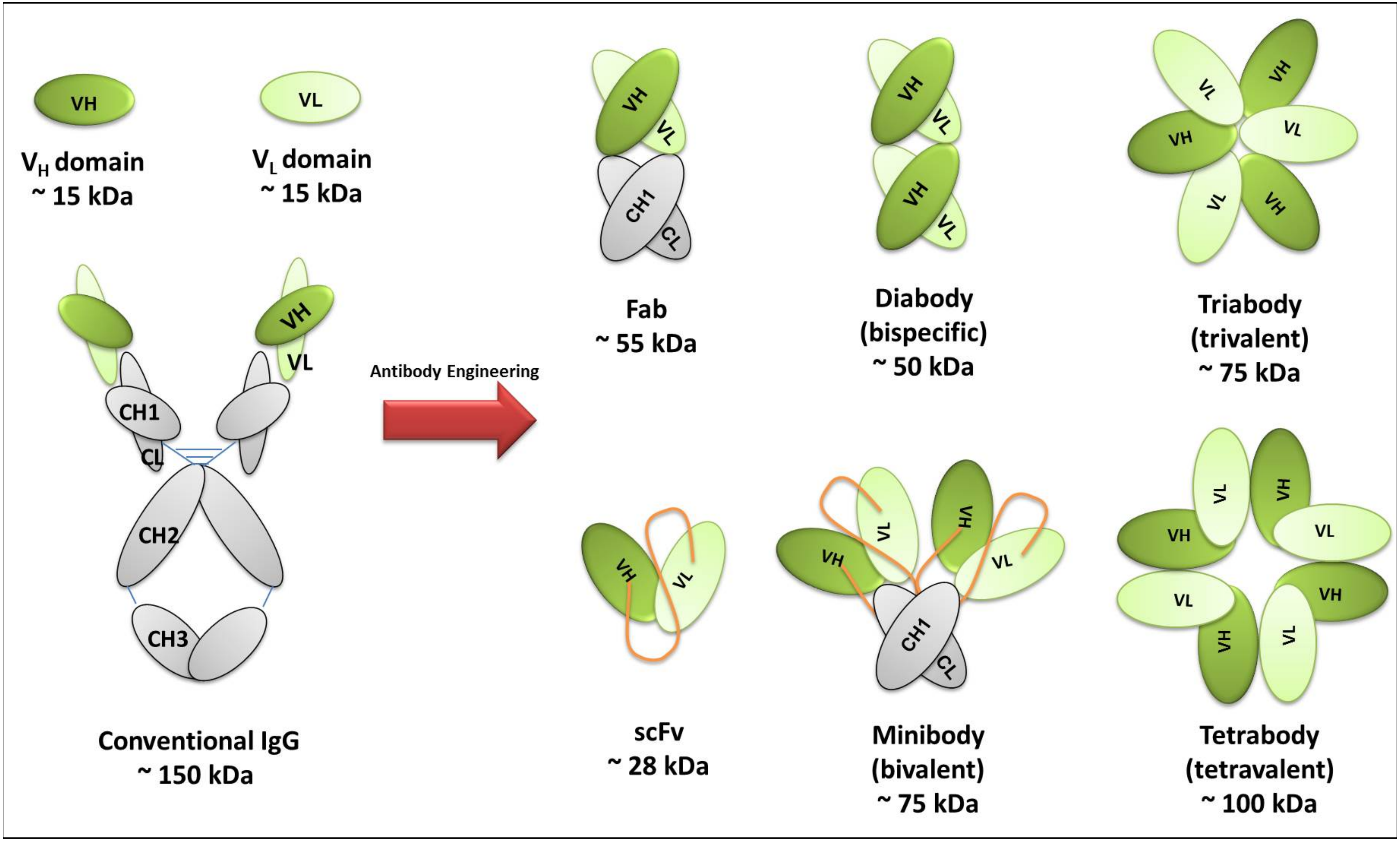

Figure S1. Schematic representation of conventional antibody IgG together with its engineered recombinant antibody fragments, including Fab fragment, scFv monomer, dimer (diabody), trimer (triabody) and tetramer (tetrabod) with linkers represented by an orange line. Minibodies are shown as two scFv modules joined by two C domains.
